# Assessment of remifentanil for rapid sequence induction and intubation in patients at risk of pulmonary aspiration of gastric contents compared to rapid-onset paralytic agents: study protocol for a non-inferiority simple blind randomized controlled trial (the REMICRUSH study)

**DOI:** 10.1186/s13063-021-05192-x

**Published:** 2021-03-30

**Authors:** Nicolas Grillot, Matthias Garot, Sigismond Lasocki, Olivier Huet, Pierre Bouzat, Charlène Le Moal, Mathieu Oudot, Nolwenn Chatel-Josse, Younes El Amine, Marc Danguy des Déserts, Nathalie Bruneau, Raphael Cinotti, Jean-Stéphane David, Olivier Langeron, Vincent Minville, Martine Tching-Sin, Elodie Faurel-Paul, Céline Lerebourg, Delphine Flattres-Duchaussoy, Alexandra Jobert, Karim Asehnoune, Fanny Feuillet, Antoine Roquilly

**Affiliations:** 1 Université de Nantes, CHU Nantes, Pôle Anesthésie-Réanimation, Service d’Anesthésie Réanimation Chirurgicale, Hôtel Dieu, Nantes, F-44093 France; 2 CHU de Lille, Pole Anesthésie Réanimation, Hôpital Claude Huriez, Lille, France; 3 Université d’Angers, CHU d’Angers, Département Anesthésie Réanimation, Angers, F-49933 France; 4 Anaesthesia, and Intensive Care Unit, Brest Regional University Hospital, Brest, France; 5 Pôle d’Anesthésie-Réanimation, Hôpital Albert Michallon, BP 217, F-38043 Grenoble, France; 6 Anaesthesia and Intensive Care Unit, Le Mans Public Hospital, Le Mans, France; 7 Anaesthesia Unit, Vendée District Hospital Center, La Roche-sur-Yon, France; 8 Anaesthesia Unit, Le Confluent Private Hospital, Nantes, France; 9 Anaesthesia Unit, Valenciennes Public Hospital, Valenciennes, France; 10 Anaesthesia and Intensive Care Unit, Clermont-Tonnerre Military Hospital, Brest, France; 11 Anaesthesia and Intensive Care Unit, Lille Regional University Hospital, Lille, France; 12 CHU Nantes, Pôle Anesthésie-Réanimation, Service d’Anesthésie Réanimation Chirurgicale, Hôpital Guillaume et René Laennec, Université de Nantes, Saint-Herblain, 44800 France; 13 Hospices Civils de Lyon, Lyon Sud Regional University Hospital, Anaesthesia and Intensive Care Unit, Lyon, France; 14 Anaesthesia and Intensive Care Unit, Henri-Mondor University Hospital (AP-HP), Créteil, France; 15 Anaesthesia and Intensive Care Unit, Toulouse University Hospital, Toulouse, France; 16 Department of Pharmacy, Nantes University Hospital, Nantes, France; 17 Department of Clinical Research, Nantes University Hospital, Nantes, France; 18 Nantes University Hospital, Methodology and Biostatistics Platform, Department of Clinical Research, Nantes, France; 19 Nantes University, INSERM, SPHERE U1246, Nantes, France

**Keywords:** Rapid sequence induction, Full stomach patient, Remifentanil, Paralytic agents, Succinylcholine, Rocuronium, Tracheal intubation

## Abstract

**Background:**

Rapid-onset paralytic agents are recommended to achieve muscle relaxation and facilitate tracheal intubation during rapid sequence induction in patients at risk of pulmonary aspiration of gastric contents. However, opioids are frequently used in this setting. The study’s objective is to demonstrate the non-inferiority of remifentanil compared to rapid-onset paralytic agents, in association with an hypnotic drug, for tracheal intubation in patients undergoing  procedure under general anesthesia and at risk of pulmonary aspiration of gastric contents.

**Methods:**

The REMICRUSH (Remifentanil for Rapid Sequence Induction of Anaesthesia) study is a multicenter, single-blinded, non-inferiority randomized controlled trial comparing remifentanil (3 to 4 μg/kg) with rapid-onset paralytic agents (succinylcholine or rocuronium 1 mg/kg) for rapid sequence induction in 1150 adult surgical patients requiring tracheal intubation during general anesthesia. Enrolment started in October 2019 in 15 French anesthesia units. The expected date of the final follow-up is October 2021. The primary outcome is the proportion of successful tracheal intubation without major complications. A non-inferiority margin of 7% was chosen. Analyses of the intent-to-treat and per-protocol populations are planned.

**Discussion:**

The REMICRUSH trial protocol has been approved by the ethics committee of The Comité de Protection des Personnes Sud-Ouest et Outre-Mer II and will be carried out according to the principles of the Declaration of Helsinki and the Good Clinical Practice guidelines. The results of this study will be disseminated through presentations at scientific conferences and publications in peer-reviewed journals. The REMICRUSH trial is the first randomized controlled trial powered to investigate whether remifentanil with hypnotics is non-inferior to rapid-onset paralytic agents with hypnotic in rapid sequence induction of anesthesia for full stomach patients considering successful tracheal intubation without major complication.

**Trial registration:**

ClinicalTrials.gov NCT03960801. Registered on May 23, 2019.

**Supplementary Information:**

The online version contains supplementary material available at 10.1186/s13063-021-05192-x.

## Administrative information

**Table:** 

Title {1}	Assessment of remifentanil for rapid sequence induction and intubation in patients at risk of pulmonary aspiration of gastric contents compared to rapid-onset paralytic agents: study protocol for a non-inferiority simple blind randomized controlled trial (The REMICRUSH study)
Trial registration {2a and 2b}.	ClinicalTrials.gov, trial registration NCT03960801, https://clinicaltrials.gov/ct2/show/NCT03960801?term=NCT03960801&draw=2&rank=1 First Posted : May 23, 2019 Last Update Posted : Jun 24, 2020
Protocol version {3}	Version 3.0, 03-08-2020.
Funding {4}	The trial is supported by a grant from the French Ministry of Health (PHRCI 2018, API18/N/015)
Author details {5a}	Nicolas Grillot - CHU Nantes Hôtel-Dieu Matthias Garot - CHU de Lille Claude Huriez Sigismond Lasocki - CHU d’Angers Olivier Huet – CHRU de Brest Pierre Bouzat - CHU de Grenoble Charlène Le Moal – CH du Mans Mathieu Oudot – CHD La Roche-sur-Yon Nolwenn Chatel-Josse – Hôpital Privé du Confluent Younes El Amine – CH de Valencienne Marc Danguy des Déserts – HIA Clermont-Tonnerre Nathalie Bruneau – CHU de Lille Salengro Raphael Cinotti - CHU Nantes Laennec, Jean-Stéphane David - Hospices Civils de Lyon, Lyon Sud Olivier Langeron – CHU Henri-Mondor Vincent Minville - CHU de Toulouse Martine Tching-Sin – CHU de Nantes Elodie Faurel-Paul – CHU de Nantes Céline Lerebourg - CHU Nantes Alexandra Jobert – CHU de Nantes Karim Asehnoune - CHU Nantes Fanny Feuillet – CHU de Nantes Antoine Roquilly – CHU de Nantes
Name and contact information for the trial sponsor {5b}	CHU de NANTES - Direction de la Recherche 5 allée de l'île Gloriette, 44093 NANTES Cedex 1 Adresse de visite : Maison de la Recherche en Santé, 53 Chaussée de la Madeleine, 44000 Nantes. Chef de Projets Département Promotion : elodie.faurelpaul@chu-nantes.fr; Tél. : +33 (0) 2 44 76 81 44 ; Fax : +33 (0) 2 53 48 28 36
Role of sponsor {5c}	The Nantes University Hospital has no role in the design or conduct of the study, the data analysis, the writing of the manuscript, or the decision to submit it. The sponsor ensures the funding of the study and subscribes to an insurance policy covering the pecuniary consequences of its civil liability under French legislation.

## Introduction

Pulmonary aspiration of gastric contents is a severe complication related to general anesthesia, whose incidence increases in patients with a full stomach. Rapid sequence induction is recommended for patients at risk of pulmonary aspiration of gastric contents (for example, emergency procedure, bowel obstruction, obese patients, or severe gastroesophageal reflux). Standard rapid sequence induction relies on the combination of a rapid hypnotic drug with a rapid-onset paralytic agent. In this setting, succinylcholine is recommended as the rapid-onset paralytic agent of first intention [1]. However, several severe adverse events are frequently reported with this drug, especially anaphylactic reaction, extended neuromuscular block, malignant hyperthermia, and severe hyperkalemia [2]. In case of counterindications of succinylcholine use, a high-dose of rocuronium, another rapid-onset paralytic agent, is recommended [3]. Unfortunately, rocuronium causes as many anaphylactic events as succinylcholine (1 anaphylactic event for 3000 uses) and induces a protracted neuromuscular blockade which frequently exceeds the duration of the surgical procedure [4]. Despite international guidelines, and probably due to safety concerns, rapid-onset paralytic agents are only used in 31 to 55% of rapid sequence induction [5–8].

When rapid-onset paralytic agents are not used for rapid sequence induction, opioids are frequently associated with hypnotics during the intubation procedure. Remifentanil, which is the opioid with the shortest delay and duration of action [9], has been proposed as an alternative to paralytic agents during rapid sequence induction. Several studies have shown that the use of remifentanil is associated with similar conditions of tracheal intubation and fewer hemodynamic reactions compared with neuromuscular blockade agents during scheduled surgery [10–14]. However, the risk of major complications during rapid sequence induction performed with remifentanil has not yet been thoroughly compared to rapid-onset paralytic agents.

We hypothesized that remifentanil associated with a hypnotic drug is not inferior to rapid-onset paralytic agents with a hypnotic drug for rapid sequence intubation for patients at risk of pulmonary aspiration of gastric contents and requiring general anesthesia for surgical procedure.

We thus designed the REMICRUSH (Remifentanil for Rapid Sequence Induction of Anaesthesia) trial, which is a multicenter, single-blinded, non-inferiority randomized controlled trial comparing remifentanil (3 to 4 μg/kg) with rapid-onset paralytic agents (succinylcholine or rocuronium 1 mg/kg) for rapid sequence induction in 1150 adult patients at risk of pulmonary aspiration of gastric contents needing orotracheal intubation under general anesthesia.

## Methods and design

### Hypothesis

The incidence of major complications during the tracheal intubation of patients at risk of pulmonary aspiration of gastric contents is not increased by remifentanil use, as a replacement for rapid-onset paralytic agents (succinylcholine or rocuronium), in intravenous rapid sequence induction and intubation.

### Research questions

1. Is remifentanil, in association with a hypnotic, non-inferior to rapid-onset paralytic agents considering successful tracheal intubation without major complications?
2. Does remifentanil use in rapid sequence induction allow similar conditions for tracheal intubation in patients at risk of pulmonary aspiration of gastric contents than rapid-onset paralytic agents?
3. Does remifentanil use in rapid sequence induction reduce the risk of postoperative respiratory complications?

### Design

The REMICRUSH (Remifentanil for Rapid Sequence Induction of Anaesthesia) trial is an investigator-initiated, multicenter, single-blinded, non-inferiority randomized controlled clinical trial.

### Ethics

The Comité de Protection des Personnes Sud-Ouest et Outre-Mer II (France) approved the study protocol (version 1.1, July 4, 2019). Patients provide written consent for participation when possible. Patients are eligible to be enrolled without legal surrogate consent if they cannot express consent due to the emergency situation and if next of kin cannot be informed in the maximal delay for inclusion. Patients who recover sufficient capacity to provide consent will be asked to consent to continue participation in the trial (model consent form and other related documentation given to participants and authorized surrogates are available in Additional file 6; in French). As we have not planned any further ancillary studies, no additional consent provisions for the collection and use of participant data and biological samples have been provided.

The REMICRUSH study is conducted under the declaration of Helsinki and is registered on May 2019 at http://clinicaltrials.gov/ with trial registration NCT03960801 (Supplemental Table S1).

## Methods

### Study population

Patients, aged from 18 to 80 years old, seen by investigators in planned anesthesia consultations or during their admission to the operating room and who will have a surgical or interventional procedure under general anesthesia with tracheal intubation and considered at risk of pulmonary aspiration of gastric contents may be offered to participate to the study. The risk of pulmonary aspiration of gastric contents was defined by at least one of the following criteria: pre-operative fasting period less than 6 h, occlusive syndrome, functional ileus, vomiting episode within the last 12 h, orthopedic trauma within the last 12 h, medical history of symptomatic gastroesophageal reflux or hiatus hernia or gastroparesis or dysautonomia or gastroesophageal surgery with sphincter dysfunction.

Patients who met any of the following criteria will be excluded from the study: pregnant women, contraindication to any rapid-onset paralytic agent (known allergy to rapid muscle relaxant drugs, personal or family history of known malignant hyperthermia, congenital muscular dystrophy, myasthenia, a known congenital deficit in plasma pseudocholinesterase), intended use of etomidate as hypnotic to induce anesthesia, prediction of impossible tracheal intubation, pre-operative respiratory failure (SpO_2_ < 95%), pre-operative hemodynamic failure (mean arterial pressure under 65 mmHg or use of vasopressor), patient in cardiac arrest, patient under justice protection, and the absence of affiliation with French social security system.

The modalities for obtaining patient consent by Investigating Physicians are described in the “Ethics” section.

### Setting

The study involves 15 French anesthesia units from university and non-university hospitals, each center caring for emergency and non-emergency anesthetic procedure (by alphabetic order: Angers University Hospital, Brest University Hospital, Clermont-Tonnerre Military Hospital, Grenoble University Hospital, La Roche-sur-Yon Public Hospital, Le Confluent Private Hospital, Le Mans Public Hospital, Lille Huriez University Hospital, Lille Salengro University Hospital, Lyon Sud University Hospital, Nantes Hotel-Dieu University Hospital, Nantes Laennec University Hospital, Toulouse Purpan University Hospital, Toulouse Rangueil University Hospital, Valenciennes Public Hospital).

### Treatment allocation

**Fig. 1:**
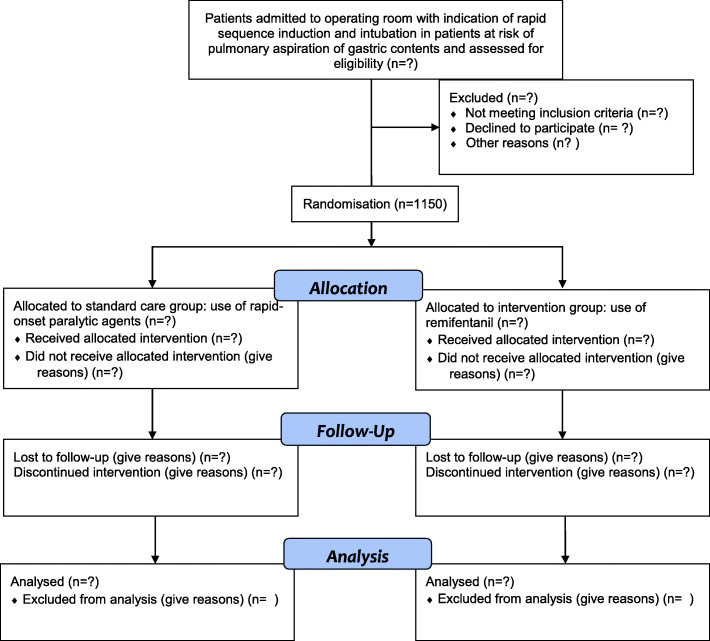
CONSORT diagram of the Remifentanil for Rapid Sequence Induction of Anaesthesia (REMICRUSH) trial illustrating the randomization and flow of patients in the study

Randomization was performed through a secure web-based randomization system. The randomization list was generated by a statistician, not involved in determining the eligibility or the assessment of outcomes. The sequences were generated using the PROC PLAN procedure from SAS, version 9.4 (SAS Institute), and the statistician was the only one who knows size of the randomization blocks. Patients are randomized to the remifentanil (intervention group) or the rapid-onset paralytic agent (control group) in fixed blocks, in a 1/1 ratio, with stratification based on the type of laryngoscopy (direct vs. indirect video-laryngoscope, which is a risk factor of successful intubation at the first attempt) and on digestive occlusive syndrome (yes or no, which is a significant risk of gastric inhalation) (Fig. 1).

### Masking protocol

It is not possible to blind local investigators to allocation as it is obvious clinically which patients are receiving rapid-onset paralytic agents. Patients are blinded to the group allocation as well as the data analysts.

### Procedures

**Fig. 2:**
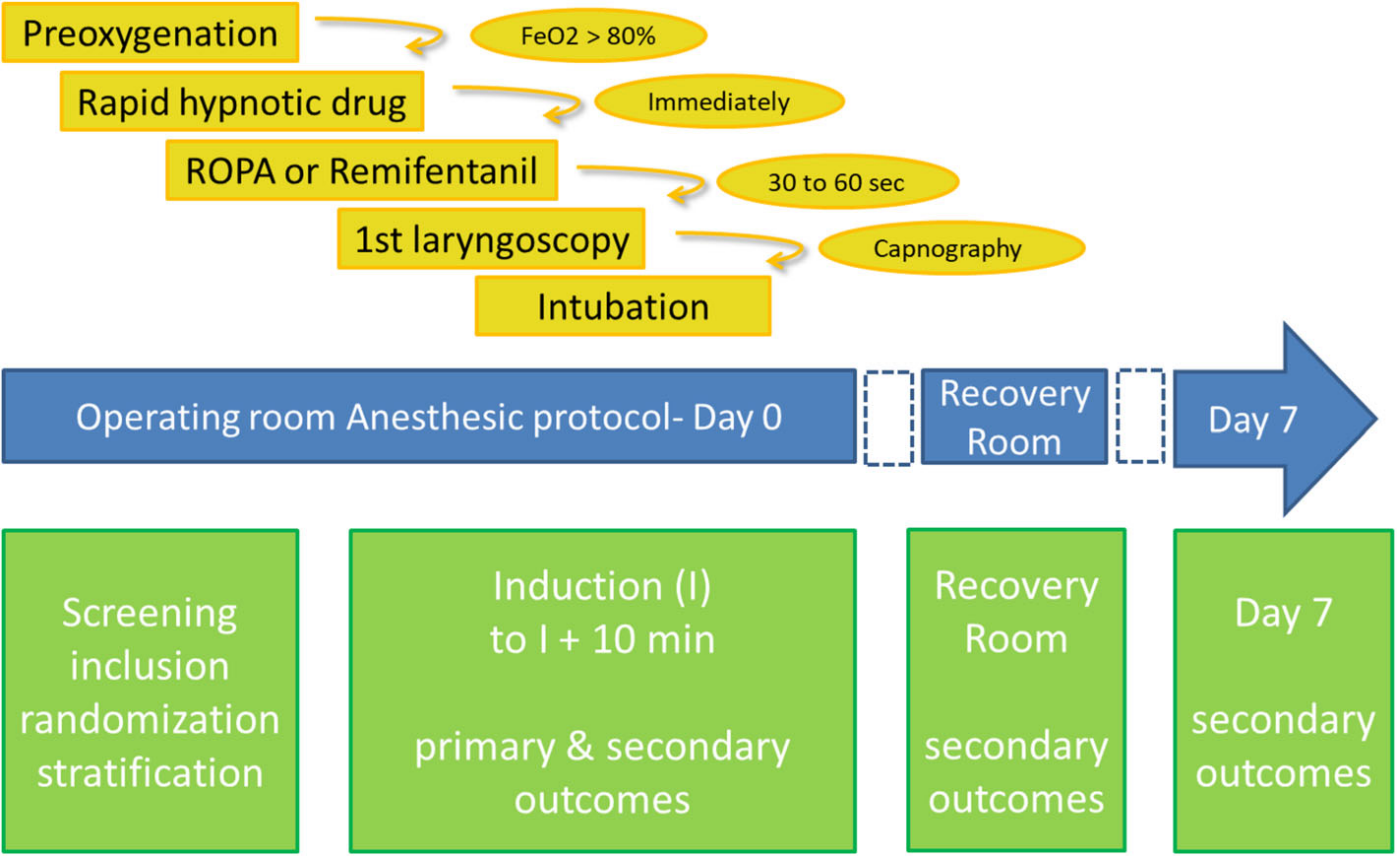
Data collection timeline for the REMICRUSH study. FeO2, fractional expired oxygen concentration, ROP, Rapid-Onset Paralytic Agent

The choice of hypnotics is at the physician’s discretion with dosage based on estimated patient weight and height. The injection of the hypnotic is followed immediately by an intravenous bolus of either remifentanil (3 to 4 μg/kg) in the intervention group or rapid-onset paralytic agent (1 mg/kg of succinylcholine or 1 mg/kg of rocuronium at the physician’s discretion) in the control group, with weight adjustment according to the body mass index (Supplemental Table S2). It is recommended to perform the tracheal intubation 30 to 60 s after administering the paralytic agent or the remifentanil. In the rapid-onset paralytic agent group, no opioid is injected before the tracheal intubation. In case of difficult unplanned intubation, the physicians are free to use a rapid-onset paralytic agent or remifentanil as rescue therapies (Fig. 2).

### Standard care

Anesthesiologists are asked to follow the French guidelines for sedation and analgesia in the operating room. A trained anesthesiologist performs the tracheal intubation (> 4 semesters of training in anesthesiology for residents) or nurses specialized in anesthesiology (> 2 years of practice). In both groups, sedation induction is performed after preoxygenation (target FeO_2_ > 80%). The choice of laryngoscopes’ device (single-use Macintosh metallic laryngoscope blades (size 3 or 4) or video laryngoscopy) is at the physician’s discretion defined before randomization (criteria of stratification of the randomization). The uses of alternative airway procedures (for instance, stylet, backward, upwards, and rightwards pressure “BURP” maneuver or Sellick maneuver) are allowed. In case of major and minor complications associated with the study intervention, general anesthesia, or surgical procedure, post-trial care will comply with national and local protocols as appropriate. Especially in case of first intubation failure, an alternative procedure including the use of video laryngoscopy, gum elastic bougie, intubating laryngeal mask airway, or trans-cricothyroid oxygenation technique is recommended under the French guidelines on difficult airway management [15]. Hypotension is managed via fluid resuscitation with crystalloids and ephedrine, and prolonged hypotension is treated with continuous intravenous catecholamine administration [16].

### Protocol drop-out

Patients not receiving the attributed drugs or receiving the other study drug as rescue therapy for non-predicted difficult intubation will be kept in analysis and remain analyzed with their randomized group.

### Study endpoints

The primary endpoint is the proportion of patients with successful tracheal intubation without any significant complications up to 10 min after induction of anesthesia. Major complications during the first 10 min after induction of anesthesia are defined as the failure of tracheal intubation at the first attempt, pulmonary aspiration of gastric contents, oxygen desaturation < 95%, severe hemodynamic instability (mean arterial pressure (MAP) ≤ 50 mmHg or ≥ 110 mmHg for more than 3 min), severe arrhythmia (i.e., requiring pharmacological or electrical intervention or lasting more than 30 s), ventricular fibrillation or cardiac arrest, and anaphylactic reaction grade III or IV in HAS classification.

Secondary endpoints which will be compared between the two study groups are as follows:

In the operating theater: median intubation quality score (IDS-3 score) values [17], Cormack-Lehane (Supplemental Figure S1) and median POGO score values [18], percentage of patients requiring tracheal intubation alternative technique, median time between the administration of the hypnotic (start of anesthetic induction) and tracheal intubation (defined as the 6th capnography curve), median minimal value of SpO_2_ value within the first 10 min after anesthesia induction, percentage of patients with moderate desaturation (SpO_2_ ≤ 95%) or severe (SpO_2_ < 80%) within the first 10 min after anesthesia induction, percentage of patients with a severe hemodynamic disorder within the first 10 min after anesthesia induction (heart rate lower than 45 beats per minute (bpm) and/or higher than 110 bpm and/or a systolic blood pressure (SBP) lower than 80 mmHg and/or a SBP higher than 160 mmHg and/or a MAP of less than 55 mmHg and/or a MAP higher than 100 mmHg), frequency of patient with dental or tracheal injury during general anesthesia, frequency of patients with an allergic event of HAS 2013 grade I or II during general anesthesia, and the median doses of vasopressors used after induction,

In the recovery room: postoperative Sore Throat Score median values [19], percentage of patients with nausea, vomiting, aspiration of gastric contents, percentage of patients with laryngeal dyspnea, percentage of patients with SpO_2_ ≤ 92, percentage of patients with SpO_2_ < 80%, percentage of patients requiring post-extubation respiratory support, proportion of reintubation, and proportion of postoperative emergency ICU admission.

After discharge of the recovery room: percentage of patients with postoperative pneumonia diagnosed up to day 7 and defined by the presence of new or progressive infiltrate on chest X-ray or C.T. scan, associated with any of the following symptoms: onset of purulent sputum, change in the appearance of chronic sputum, fever ≥ 38 °C, hyperleukocytosis (> 12,000/mL) or leukopenia (< 4000/mL), positive blood cultures or isolation of the pathogen on sputum, tracheal aspiration or bronchoalveolar lavage, percentage of patients with acute respiratory failure occurring up to day 7 defined by the combination of a clinical picture of acute hypoxemic respiratory failure, a PaO_2_/FiO_2_ ratio < 300 mmHg, the presence of new bilateral pulmonary infiltrates on the chest X-ray and the absence of evidence of cardiac origin, intra-hospital mortality up to day 7, and survival proportion at day 7.

Proportion of adverse events will be compared between the study groups. All expected and unexpected serious adverse events, as defined as EMA grade 3 (severe), 4 (life-threatening), to 5 (lethal), will be systemically recorded on day 1 and day 7. The proportion of expected and unexpected serious adverse events will be analyzed and reported in future publications.

### Schedule of enrolment, interventions, and assessments

A schematic diagram of the REMICRUSH study is available in Supplemental Figure S2. No biological specimens would be collected as part of this trial.

### Follow-up data

Most of the data are collected during the first 10 min after induction of anesthesia. As the anesthetic procedure progresses, the data collection sheet is completed in the recovery room and attached to the medical record. Data are also reported in a centralized electronic case report form. The duration of patient follow-up is 7 days. In the event of earlier discharge from the hospital, patients are considered free from complications for the days out of the hospital.

### Data collection and checking

An online case-report form is used for the collection of data. Blinded and patient identifiable data are stored separately in secure databases. The investigation center stores all identifiable patient data. Research assistants from the promoting center work closely with local investigators to obtain data as complete and accurate as possible.

### Study monitoring

The sponsor (Nantes University Hospital) is in charge of data monitoring. Site staff will be available to facilitate the monitoring visits and ensure that all required documentation is available for review. Study initiation visits have been carried out at all sites before recruitment begins at that site. During regular monitoring visits realized throughout the trial, an independent research assistant will carry out Source Data Verification of trial data, verify informed consent forms, and ensure the Investigator Site Files’ completeness.

### Study oversight

Study sponsor is the Nantes University Hospital (5 allée de l’île Gloriette, 44,000 Nantes, drc-nantes@chu-nantes.fr). Experienced research staff monitored the study for quality the integrity of data in all the participating centers. Serious adverse events and unexpected related or possibly related severe events are reported to the sponsor within 7 days.

The sponsor appoints an independent data and safety monitoring board (DSMB). The DSMB is composed up of 3 people with no connection to the research, including one co-author of the French Guidelines on muscle relaxants and reversal in anesthesia [20], one pharmacovigilance specialist, and one methodologist/biostatistician. Every 400 inclusions, the DSMB reviews safety issues as the study progresses. The DSMB makes recommendations to the sponsor about the continuation, modification, or termination of the research. The recommendations that the DSMB can make are:

- To continue the research with no modifications
- To continue the research with a modification to the protocol or the monitoring of subjects
- To temporarily halt inclusions
- To permanently terminate the research in light of severe adverse reactions.

The sponsor can stop trial recruitment on the advice of the DSMB in case of safety concerns.

The Scientific Committee, composed of Dr. Grillot (M.D.), Pr. Roquilly (M.D, Ph.D.), and F. Feuillet (Ph.D) approves the main study protocol and any amendments to monitor and supervise the trial concerning its primary and secondary objectives, to review relevant information from additional sources, to consider the recommendations of the DSMB, and to resolve issues raised by the trial coordinating centers.

Dr. Grillot, as coordinating investigator, is responsible for the governance of the study with the Scientific Committee, the Study Sponsor, and the DSMB.

### Roles of the sponsor and the funder

The sponsor and of the funder had no role in the design or conduct of the study, the data analysis, the writing of the manuscript, or in the decision to submit the manuscript.

### Sample size

The proportion of successful tracheal intubation without any major complications is the primary outcome. In previous studies, the proportion of rapid sequence induction of general anesthesia with a rapid-onset paralytic agent without major complications ranged between 70 and 92%, depending on the studies and definitions [19–21]. It is also recommended to set non-inferiority margins lower than a 10% relative difference between the intervention and control groups [22]. Assuming an 80% rate of tracheal intubation without major complication in the rapid-onset paralytic agent group, and given the potential benefit of avoiding the use of rapid paralytic agents, we deemed it medically acceptable to set the non-inferiority margin to an absolute difference of 7% (8.75% relative difference). We thus calculated that a total of 1150 patients (575 patients per group) was needed to demonstrate the non-inferiority of the intervention with a statistical power of 80% an alpha risk of 2.5.

### Pre-planned primary analysis

The statistical analysis will be carried out under the current recommendations for non-inferiority trial analysis [23].

The analysis of the primary outcome will be undertaken on the intention to treat principle including all randomized patients, and under the “per-protocol” principle, including all randomly assigned patients except those who withdrew consent for the use of all trial data, those who did not meet the inclusion criteria, those who did not receive the allocated study treatment, and those without data for the primary outcome [22, 23]. To comply with international recommendations on non-inferiority trials [24], the analysis based on the intention-to-treat principle and the data set for the per-protocol analysis will have equal importance to lead similar to a robust interpretation.

For the primary endpoint (proportion of successful tracheal intubation without major complications), the proportion difference between the two groups and the two-sided 95% confidence interval will be estimated. Under our assumptions, the remifentanil group will be considered non-inferior if the upper limit of the two-sided 95% confidence interval of absolute proportion difference does not exceed 7% (non-inferiority margin). The confidence interval will be estimated by a logistic model considering stratification on the existence or not of an occlusive syndrome and the type of equipment used for intubation. As the primary endpoint is identified within 10 min of anesthetic induction and death is included in the endpoint, a small amount of missing data will be expected. Multiple imputations methods based on demographic criteria and available data will be used in case of missing data. If non-inferiority is shown for the primary endpoint (on “intention-to-treat” and “per-protocol” populations), a superiority test will be assessed.

Preplanned subgroup analyses will be carried out on the primary outcome, following the same methodology:

- Randomization stratum
  *Type of equipment used for intubation (Macintosh laryngoscope vs. video laryngoscope).
  *Primary risk factor for pulmonary aspiration of gastric contents (occlusive syndrome vs. other causes)
- Age groups (18–40 years; 40–60 years; 60–80 years)
- Mallampati score (I and II vs. III and IV)
- Standard of care (urgent or non-urgent)
- Body mass index greater than 30 kg/m^2^ (yes vs. no)
- ASA Score (1–2 vs. 3–4)
- Choice of propofol as hypnotic (propofol vs. others)

For secondary endpoints, the two groups will be compared with the use of logistic regression models for binary data (postoperative pneumonia, postoperative respiratory distress), with the use of linear regression models for continuous data (heart rate, systolic blood pressure) and with the use of Poisson regression models for count data (number of episodes of allergic manifestation, number of episodes of desaturation). All analyses will be adjusted for stratification factors and centers as random effect.

Continuous variables will be presented as mean and S.D.s (as median and quartiles, otherwise). Categorical data will be presented as exact numbers and percentages.

The DSMB will periodically conduct safety analyses, as described in the “Study oversight” section.

### Data sharing

The coordinating investigator will have access to the final trial data set. Patient-level data and full dataset and statistical code will be available upon request to the corresponding author. Consent for data sharing was not obtained, but the presented data are anonymized, and risk of identification is low, and the potential benefits of sharing these data outweigh the potential harms.

### Protocol amendments

Requests for substantial modifications will be sent by the promoter for authorization to the ANSM and authorization/information to the ethics committee under the legislation. The modified protocol will have to be the subject of a dated updated version. The patient information and consent forms will be modified if necessary.

### Authorship rules

For further publication, the coordinating investigator (NG), the statistician-methodologist (F.F.), and the principal investigator of the study (A.R.) will be the first, second last, and last authors, respectively. The second authorship place and subsequent ones will be allocated to the participating centers according to the total number of patients included in the trial. A minimal number of 60 inclusions is deemed necessary to gain authorship.

## Discussion

The REMICRUSH trial is the first randomized controlled study powered to investigate remifentanil efficiency for rapid sequence induction in adults compared with rapid-onset paralytic agents.

Induction of anesthesia in a non-fasting patient is primarily intended to limit the risk of major complications, including pulmonary aspiration of gastric contents [25]. Therefore, pharmacological agents should provide good tracheal intubation conditions: have a short onset of action and a short duration. Current recommendations support the use of succinylcholine, which may reduce the risk of complications related to emergency intubation [15]. Due to concerns about tolerance, up to 50% of rapid sequence intubation are made without succinylcholine currently. The use of rocuronium as an alternative does not eliminate the risk of anaphylaxis, and its pharmacokinetic profile may be an additional issue.

Because of its pharmacokinetic characteristics and the absence of anaphylaxis risk, remifentanil is commonly used for anesthetic intubation. In the case of non-inferiority demonstrated in our study, the use of remifentanil may represent an alternative to rapid-onset paralytic agents, notably for physicians who do not use rapid-onset paralytic agents and for patients with negative risk-benefit balance for rapid-onset paralytic agents.

We choose to use the frequency of tracheal intubation without major complications as a clinically relevant criterion that covers the main complications during rapid sequence induction. There are no recommendations for selecting the primary endpoint in studies evaluating the modalities of rapid sequence induction in the operating room. Routine sequence intubation has excellent efficacy, close to 97% intubation at the first attempt [26]. The feasibility of intubation during anesthesia induction with remifentanil without a paralytic agent has already been demonstrated [10–14]. Therefore, the main criterion for choosing between the rapid sequence induction protocols is the frequency of severe complications after intubation. We aim to demonstrate the non-inferiority of remifentanil on the incidence of uncomplicated intubation. We defined uncomplicated intubation by a composite criterion adapted from studies evaluating emergency intubation in anesthesia and resuscitation [27–29] and based on the major complications associated with rapid sequence induction of anesthesia.

## Trial status

Protocol version number: Version 3.0, 03-08-2020.

Protocol date: July 4, 2019

Recruitment start date: October 9, 2019

Planned recruitment end date: October 2021

The trial has already achieved many milestones. Sponsorship has been agreed: the trial is sponsored by the French ministry of health. Insurance for non-negligent harm has been provided by the University Hospital of Nantes (CHU Nantes, France). Research ethics committee approval was obtained in July 2019. It is registered with the American registry of trials (https://clinicaltrials.gov/ NCT03960801). Inclusions started on October 9, 2019. Due to the pandemic of COVID-19 pneumonia, the study has been suspended from March 20, 2020, to May 4, 2020, after the inclusion of 340 patients and before the first DSMB meeting. The recruitment process was resumed but we remain vigilant regarding the epidemiological evolution of COVID-19 pandemic.

## Supplementary Information

**Additional file 1: Supplemental Figure S1.** Cormack-Lehane classification system.

**Additional file 2: Supplemental Figure S2.** SPIRIT Figure of the REMICRUSH study.

**Additional file 3: Supplemental Figure S3.** SPIRIT Checklist of the REMICRUSH study Manuscript.

**Additional file 4: Supplemental Table S1.** WHO Trial Registration Dataset.

**Additional file 5: Supplemental Table S2.** Weight adaptation dose for remifentanil and rapid-onset paralytic agents in the REMICRUSH study.

**Additional file 6.** Model consent form and other related documentation given to participants and authorized surrogates (in French).

## Data Availability

As the sponsor of the trial, the Nantes University Hospital is the owner of the data. After the trial, the Nantes University Hospital will provide a copy of the trial dataset to the trial investigators (NG) and the trial statistician (FF) to fully participate in developing the leading publication.

## Abbreviations

ASA: Physical status score of the American Society of Anesthesiologists
ANSM: Agence nationale de sécurité du médicament et des produits de santé (National Agency for the Safety of Medicines and Health Products)
BMI: Body mass index
bpm: Beats per minute
COVID-19: Coronavirus disease 2019
DSMB: Data and safety monitoring board
ICU: Intensive care unit
MAP: Mean arterial pressure
SBP: Systolic blood pressure
