# Supplementary material for: Assessment of remifentanil for rapid sequence induction and intubation in patients at risk of pulmonary aspiration of gastric contents compared to rapid-onset paralytic agents: study protocol for a non-inferiority simple blind randomized controlled trial (the REMICRUSH study)
Source: Trials. 2021 Mar 30;22:237. doi: 10.1186/s13063-021-05192-x (PMC8009075; doi:10.1186/s13063-021-05192-x)
Supplement: Supplementary file 1 — Additional file 1: Supplemental Figure S1. Cormack-Lehane classification system. [file 13063_2021_5192_MOESM1_ESM.doc]

*
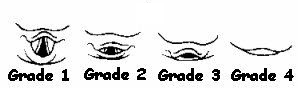
*

Supplemental Figure S 1, Cormack-Lehane classification system

*The Cormack-Lehane system classifies views obtained by direct laryngoscopy based on the structures seen. Grade 1: Full view of glottis, Grade 2 : Partial view of glottis or only posterior extremity of glottis seen or only arytenoid cartilages, Grade 3: Only epiglottis seen, none of glottis seen, Grade 4: Neither glottis nor epiglottis seen*
