# Supplementary material for: Assessment of remifentanil for rapid sequence induction and intubation in patients at risk of pulmonary aspiration of gastric contents compared to rapid-onset paralytic agents: study protocol for a non-inferiority simple blind randomized controlled trial (the REMICRUSH study)
Source: Trials. 2021 Mar 30;22:237. doi: 10.1186/s13063-021-05192-x (PMC8009075; doi:10.1186/s13063-021-05192-x)
Supplement: Supplementary file 2 — Additional file 2: Supplemental Figure S2. SPIRIT Figure of the REMICRUSH study. [file 13063_2021_5192_MOESM2_ESM.doc]

Supplemental Figure S2. SPIRIT Figure of the REMICRUSH study.

|  | **STUDY PERIOD** | | | | | |
| --- | --- | --- | --- | --- | --- | --- |
|  | **Enrolment** | **Allocation** | **Post-allocation (OR)** | | | **Close-out** |
| **TIMEPOINT** | ***AC*** | **Day 0 (OR)** | ***IA*** | ***IA +10 min*** | ***RR*** | ***Day 7*** |
| **ENROLMENT:** |  |  |  |  |  |  |
| **Eligibility screen** | X |  |  |  |  |  |
| **Informed consent or emergency procedure** | X |  |  |  |  |  |
| **Allocation** |  | X |  |  |  |  |
| **INTERVENTIONS:** |  |  |  |  |  |  |
| ***Primary Outcome*** |  |  | X | X |  |  |
| ***Secondary Outcomes*** |  |  | X | X | X | X |
| **ASSESSMENTS:** |  |  |  |  |  |  |
| ***Efficiency and/or safety during RSII*** |  |  | X | X |  |  |
| ***Efficiency and/or safety in RR*** |  |  |  |  | X |  |
| ***Efficiency and/or safety at D7*** |  |  |  |  |  | X |
| ***Adverse Event*** |  |  | X | X | X | X |

AC: Anesthetic Consultation; OR: Operating Room; IA: Induction of Anesthesia; RR: Recovery Room; RSII: Rapid Sequence of Induction and Intubation
