# Supplementary material for: Assessment of remifentanil for rapid sequence induction and intubation in patients at risk of pulmonary aspiration of gastric contents compared to rapid-onset paralytic agents: study protocol for a non-inferiority simple blind randomized controlled trial (the REMICRUSH study)
Source: Trials. 2021 Mar 30;22:237. doi: 10.1186/s13063-021-05192-x (PMC8009075; doi:10.1186/s13063-021-05192-x)
Supplement: Supplementary file 4 — Additional file 4: Supplemental Table S1. WHO Trial Registration Dataset. [file 13063_2021_5192_MOESM4_ESM.docx]

**Supplemental Table S 1. WHO Trial Registration Dataset**

| **Data category** | **Information** |
| --- | --- |
| Primary registry and trial identifying number | ClinicalTrials.gov NCT03960801 |
| Date of registration in primary registry | May 23, 2019 |
| Secondary identifying numbers | RC19_0055 ; 2019-000753-31 ( EudraCT Number ) |
| Source(s) of monetary or material support | Funded by the French Ministry of Health, PHRCI 2018 |
| Primary sponsor | Nantes University Hospital |
| Secondary sponsor(s) | Non applicable |
| Contact for public or scientific queries | Nicolas GRILLOT, M.D.,  Université de Nantes, CHU Nantes, Pôle Anesthésie-Réanimation, Service d’Anesthésie Réanimation Chirurgicale, Hôtel Dieu, Nantes, F-44093 France  E-mail : nicolas.grillot@chu-nantes.fr  Phone (+33)253482224, Fax (+33)240087382 |
| Public title | Assessment of Remifentanil for Rapid Sequence Induction and Intubation in Full Stomach Patient Compared to Muscle Relaxant |
| Scientific title | Assessment of Remifentanil for Rapid Sequence Induction and Intubation in Full Stomach Patient Compared to Muscle Relaxant. A Non-inferiority Simple Blind Randomized Controlled Trial |
| Countries of recruitment | France |
| Health condition(s) or problem(s) studied | Anesthetic Intra-tracheal Intubation, patients at risk of pulmonary aspiration of gastric contents |
| Intervention(s) | Drug: Remifentanil group: bolus intravenous injection of 3 to 4µg/kg of remifentanil after hypnotic administration for a crush anesthetic induction  Drug: neuromuscular blockade group : Bolus intravenous injection of 1mg/kg of Succinylcholine or Rocuronium after hypnotic administration for a crush anesthetic induction |
| Key inclusion and exclusion criteria | Inclusion Criteria: male or female; aged from 18 to 80 years old ; surgery requiring general anaesthesia with oro-tracheal intubation in Rapid sequence induction ; aspiration risk defined as : fasting < 6h00, digestive occlusion, functional ileus, vomiting < 12h00, orthopaedic trauma < 12h00, severe gastric reflux, gastroparesis and/or dysautonomia and or gastro-oesophagus surgery ; signed informed consent sheet ; or emergency procedure if impossible  Exclusion Criteria: planned impossible intubation ; suspected/known allergy to neuromuscular blockade or remifentanil ; Neuromuscular disease forbidding neuromuscular blockade use ; Prolonged neuromuscular block former episode ; Malignant hyperthermia former episode ; Pre-operative respiratory failure (spO2< 95%) ; Pre-operative hemodynamic failure (use of vasopressor) ; cardiac arrest ; A woman of childbearing age who has an active pregnancy and/or clinical signs suggestive of an active pregnancy and/or does not have a contraceptive or contraceptive method and has had unprotected sex within 15 days of the last menstrual period ; Patients under justice protection ; Use of etomidate for anesthetic induction |
| Study type | Interventional Allocation: randomized Intervention model: parallel assignment in two groups assigned to tested drug in intervention group and to reference treatment for control group. Masking: Double (Participant, Outcomes Assessor)  Primary purpose: Supportive Care Phase III |
| Date of first enrolment | October, 2019 |
| Target sample size | 1150 |
| Recruitment status | Recruiting |
| Primary outcome(s) | Primary endpoint is the rate of tracheal intubation without major complications as defined by tracheal intubation with less than 2 laryngoscopies, no aspiration during the 10 minutes after induction, no desaturation under 95% during the 10 minutes after induction, no hypo or hypertension as defined by a MAP<50mmHg or >110mmHg, no ventricular arrhythmia involving an emergency treatment or cardiac arrest during the 10 minutes after induction, no grade III or IV anaphylactic reaction during the 10 minutes after induction |
| Key secondary outcomes | Quality and difficulty level of intubation, delay of intubation, non-major hemodynamic or respiratory complications, respiratory complications in recovery room, respiratory complications at day seven, in hospital mortality |
