# Supplementary material for: Assessment of remifentanil for rapid sequence induction and intubation in patients at risk of pulmonary aspiration of gastric contents compared to rapid-onset paralytic agents: study protocol for a non-inferiority simple blind randomized controlled trial (the REMICRUSH study)
Source: Trials. 2021 Mar 30;22:237. doi: 10.1186/s13063-021-05192-x (PMC8009075; doi:10.1186/s13063-021-05192-x)
Supplement: Supplementary file 5 — Additional file 5: Supplemental Table S2. Weight adaptation dose for remifentanil and rapid-onset paralytic agents in the REMICRUSH study. [file 13063_2021_5192_MOESM5_ESM.doc]

Supplemental Table S 2. Weight adaptation dose for remifentanil and rapid-onset paralytic agents in the REMICRUSH study

| *Body weight classification* | *Formula (unit)* | *Drug* | *References* |
| --- | --- | --- | --- |
| *Body mass index(BMI)* | *w/h(in meter)²* | *-* | *Obesity is defined as a BMI ≥ 30 kg.m-2* |
| *Total Body Weight* | *no formula* | *Succinylcholine (regardless of weight)*  *Remifentanil (BMI < 30 kg.m-2)*  *Rocuronium ( BMI < 30 kg.m-2)* | *Lemmens H. The dose of succinylcholine in morbid obesity. Anesth Analg 2006;102 :438-42* |
| *Lean Body Weight* | *Female = (9270 x w) / (8780+244xBMI)*  *Male = (9270 x w) / (6680+216xBMI)* | *Remifentanil ( BMI ≥ 30 kg.m-2)*  *Rocuronium (BMI ≥ 30 kg.m-2)* | *Janmahasatian S. Quantification of lean bodyweight. Clin Pharmacokinet.2005 ;44(10) :1051-65* |

*weight (w) in kilograms(kg), height(h), in centimeter(cm)*
