# Supplementary material for: Assessment of remifentanil for rapid sequence induction and intubation in patients at risk of pulmonary aspiration of gastric contents compared to rapid-onset paralytic agents: study protocol for a non-inferiority simple blind randomized controlled trial (the REMICRUSH study)
Source: Trials. 2021 Mar 30;22:237. doi: 10.1186/s13063-021-05192-x (PMC8009075; doi:10.1186/s13063-021-05192-x)
Supplement: Supplementary file 6 — Additional file 6. Model consent form and other related documentation given to participants and authorized surrogates (in French). [file 13063_2021_5192_MOESM6_ESM.pdf]

Évaluation du REMIFENTANIL en remplacement du curare pour l'induction anesthésique en séquence rapide chez le patient à risque d'inhalation de liquide gastrique- Étude multicentrique, prospective, contrôlée, randomisée, en simple aveugle, de non infériorité

Etude REMICrush

Promoteur : CHU de Nantes

N°EudraCT : 2019-000753-31

Madame, Monsieur,

Nous vous proposons de participer à une recherche interventionnelle nommée REMICrush. Avant de vous décider, merci de lire soigneusement les informations qui suivent. Nous sommes également là pour répondre à toutes vos questions d'ici avant que nous ne débutions votre prise en charge anesthésique.

Votre état de santé actuel nécessite une intervention chirurgicale ou endoscopique urgente sous anesthésie générale. Les risques de cette anesthésie sont minimes mais non nuls. Ils sont en partie dus aux médicaments utilisés pour vous faire dormir mais également au fait que vous ayez mangé dans les 12 heures précédent votre intervention chirurgicale (non-respect du jeun pré-opératoire).

Pour réaliser une anesthésie générale dans ce contexte, il est recommandé d'administrer deux produits par voie intraveineuse :

- un hypnotique qui induit le sommeil
- un curare (le SUXAMETHONIUM ou le ROCURONIUM) qui est un relaxant musculaire puissant servant à faciliter l'introduction rapide de la sonde d'intubation dans la trachée (ce qui servira à assurer votre oxygénation et protégera vos poumons d'une éventuelle inhalation de liquide gastrique durant l'intervention).

Les curares peuvent être responsables d'effets secondaires imprévisibles et potentiellement graves (notamment allergiques). Le REMIFENTANIL est un morphinique déjà utilisé pour l'induction anesthésique depuis plus de 10 ans et qui est de plus en plus fréquemment utilisé à la place des curares car il n'expose pas au risque d'allergie.

Nous souhaitons confirmer que le REMIFENTANIL permet la mise en place de la sonde d'intubation dans d'aussi bonnes conditions que les curares dans le contexte du non-respect du jeûn pré-opératoire.

QUEL EST L'OBJECTIF DE CETTE ETUDE

L'objectif de cette étude est de comparer l'utilisation du REMIFENTANIL à celle des curares dans l'anesthésie en urgence. Le promoteur de cette recherche est le CHU de Nantes, c'est à dire qu'il en est responsable et qu'il l'organise.

QUELS SONT LES BENEFICES ATTENDUS ET LES RISQUES POTENTIELS

Nous faisons l'hypothèse que l'utilisation du REMIFENTANIL lors de l'anesthésie générale en urgence procure des conditions d'intubation similaires aux curares, et qu'elle est mieux tolérée. En effet, bien que l'utilisation des curares pour l'intubation fasse référence depuis les années 1970, leur administration peut être responsable d'évènements indésirables imprévisibles et potentiellement graves notamment allergiques.

Le REMIFENTANIL est un morphinique utilisé depuis le début des années 2000 pour l'induction et l'entretien de l'anesthésie générale. Utilisé dans ses indications habituelles, la tolérance de ce traitement est bonne. Ses principaux effets indésirables sont ceux des morphiniques, à savoir chute de la pression artérielle, ralentissement du rythme cardiaque, ralentissement de la fréquence respiratoire, nausées, vomissements, et

dans de rares cas une rigidité thoracique. Si toutefois l'un de ces effets secondaires survenait, le traitement approprié serait mis en place immédiatement.

Si vous participez à cette recherche, et que vous recevez le REMIFENTANIL, votre risque de développer une réaction allergique grave ou un trouble métabolique (augmentation de la quantité de potassium dans le sang) pourrait être diminué en comparaison des patients qui recevront des curares mais il pourrait également être plus difficile de mettre en place la sonde d'intubation trachéale.

METHODOLOGIE, CONTRAINTES ET DUREE DE L'ETUDE

La recherche est réalisée en collaboration avec plusieurs autres centres hospitaliers en France. Il est prévu d'inclure au total 1150 patients non à jeun pour lesquels une chirurgie sous anesthésie générale en urgence est prévue.

Comme les données médicales disponibles actuellement sont insuffisantes pour affirmer notre hypothèse, l'évaluation du REMIFENTANIL se fera par comparaison avec l'injection d'un curare.

Ainsi, chaque patient qui participera à cette étude sera affecté à l'un des deux groupes de traitement par tirage au sort (randomisation): la moitié des patients recevra la procédure classique associant un hypnotique et le curare et l'autre moitié recevra la procédure à l'étude associant un hypnotique et le REMIFENTANIL. Une comparaison des deux groupes sera faite en fin d'étude.

Si la procédure intégrant le REMIFENTANIL vous était attribuée, et que lors de l'anesthésie votre médecin jugeait nécessaire d'utiliser un curare en raison d'une situation particulière, il pourra tout à fait le faire.

Différentes informations seront recueillies au cours de la procédure : pouls, tension artérielles, taux d'oxygène dans le sang, mesures des différents temps (délai de perte de conscience, durée d'intubation…) et surtout la qualité de mise en place de la sonde d'intubation.

Il n'y a pas de prise de sang supplémentaire pour cette recherche.

La durée totale de votre suivi se limitera aux 7 jours suivant votre chirurgie sans que cela ne modifie votre durée de séjour à l'hôpital.

QUE SE PASSERA-T-IL A LA FIN DE LA RECHERCHE, SI LA RECHERCHE S'ARRETE ?

La recherche peut être interrompue à tout moment:

- par les autorités de santé,
- du fait du promoteur, le CHU de Nantes : si un élément nouveau survient, l'investigateur en sera informé et il vous transmettra alors les éléments susceptibles de modifier votre participation.
- du fait de l'investigateur, pour des raisons médicales vous concernant : il peut décider à tout moment d'arrêter l'administration du produit à l'étude (par exemple à cause d'un effet secondaire ou d'une évolution de votre état de santé) et vous en informera.

Quelle que soit la raison de l'interruption, l'investigateur vous informera alors des mesures à suivre. Du fait du délai extrêmement court entre l'administration de la procédure à l'étude et son effet, l'interruption de la recherche n'impactera d'aucune manière votre prise en charge actuelle ou future.

QUE SE PASSERA-T-IL A LA FIN DE LA RECHERCHE, SI VOUS DECIDEZ D'INTERROMPRE VOTRE PARTICIPATION ?

Si vous décidez de participer à cette recherche, il s'agira d'un acte volontaire. Vous pourrez à tout moment décider d'arrêter votre participation, sans pénalité ni préjudice. Dans ce cas, vous devez informer l'investigateur de votre décision.

Quelle que soit la raison de votre interruption, aucune mesure supplémentaire ne sera prise car vous aurez déjà reçu la procédure à l'étude.

Dans tous les cas, la qualité de votre prise en charge ne sera pas diminuée.

QUELS SONT VOS DROITS PENDANT LA RECHERCHE ?

❖ SECRET PROFESSIONNEL

Le personnel impliqué dans la recherche est soumis au secret professionnel, tout comme votre médecin traitant.

Sauf avis contraire de votre part, votre médecin traitant pourra être informé de votre participation.

❖ ACCES AUX DONNEES VOUS CONCERNANT - TRAITEMENT DES DONNEES - CONFIDENTIALITE

Dans le cadre de cette recherche, un traitement informatique de vos données personnelles va être mis en œuvre : cela permettra d'analyser les résultats de la recherche et de remplir l'objectif de la recherche.

Pour cela, les données médicales vous concernant seront recueillies dans un cahier électronique (eCRF).

Afin d'assurer leur confidentialité, ces données seront identifiées par un numéro de code et vos initiales.

L'ensemble de ces données sera transmis au Promoteur de la recherche (CHU de Nantes).

Ces données pourront également, dans des conditions assurant leur confidentialité, être transmises aux autorités sanitaires habilitées.

Elles seront susceptibles d'être exploitées dans le cadre de publications ou de communications; dans ce cas, votre anonymat sera préservé.

Si vous décidez de retirer votre consentement pour participer à la recherche, les données obtenues avant que celui-ci n'ait été retiré seront utilisées. Les données recueillies après le retrait de votre consentement ne seront pas utilisées pour cette recherche et resteront destinées à l'usage strict du soin.

Conformément aux dispositions de la loi relative à l'informatique aux fichiers et aux libertés (loi modifiée du 6 janvier 1978), de la loi n° 2018-493 du 20 juin 2018 relative à la protection des données personnelles et du Règlement (UE) 2016/679 du Parlement européen et du Conseil du 27 avril 2016 relatif à la protection des personnes physiques à l'égard du traitement des données à caractère personnel et à la libre circulation de ces données (RGPD), vous disposez d'un droit d'accès, de rectification, et de limitation du traitement de vos données personnelles. Vous pouvez également porter une réclamation auprès d'une autorité de contrôle (CNIL pour la France : https://www.cnil.fr/fr/webform/adresser-une-plainte/).

Ces données pourront être utilisées lors de recherches ultérieures exclusivement à des fins scientifiques. Vous pouvez retirer votre consentement à cette utilisation ultérieure ou exercer votre faculté d'opposition à tout moment.

Vous pouvez également accéder directement ou par l'intermédiaire d'un médecin de votre choix à l'ensemble de vos données médicales en application des dispositions de l'article L 1111-7 du Code de la Santé Publique.

Vos données seront conservées tout au long de la recherche. Après la fin de la recherche, les données seront archivées pour une durée conforme aux dispositions réglementaires, puis détruites.

Pour en savoir plus ou exercer vos droits concernant vos données, voir vos contacts ci-dessous.

❖ ACCES AUX RESULTATS GLOBAUX DE LA RECHERCHE

A la fin de la recherche, et à votre demande, vous pourrez être informé(e) par l'investigateur des résultats globaux de cette recherche (dès qu'ils seront disponibles).

QUELLES SONT VOS OBLIGATIONS PENDANT LA RECHERCHE ?

- Vos obligations

Vous devez informer l'investigateur de tous les médicaments que vous prenez.

Vous devez aussi l'informer immédiatement de tout évènement ou effet indésirable éventuellement rencontré au cours de votre participation à la recherche.

- Protection sociale

Pour pouvoir participer à cette recherche vous devez être affilié(e) ou bénéficier d'un régime de sécurité sociale

- Modalites de participation a une autre recherche

Vous ne pourrez pas participer à une autre recherche interventionnelle pendant toute la durée de votre participation à la Recherche c'est-à-dire 24 heures après votre chirurgie.

QUEL EST LE CADRE REGLEMENTAIRE DE CETTE RECHERCHE ?

Cette recherche est conforme :

- Aux articles L. 1121-1 à L. 1126-12 du code de la santé publique relatifs aux recherches impliquant la personne humaine

- A la loi « Informatique et Libertés » du 6 janvier 1978 modifiée et la loi n° 2018-493 du 20 juin 2018 relative à la protection des données personnelles

- au Règlement (UE) 2016/679 du Parlement européen et du Conseil du 27 avril 2016 relatif à la protection des personnes physiques à l'égard du traitement des données à caractère personnel et à la libre circulation de ces données (RGPD)

Vous pouvez retrouver tous ces textes sur le site http://www.legifrance.gouv.fr

Conformément aux dispositions réglementaires :

- Le CHU de Nantes organise cette recherche en tant que « promoteur ». Il a souscrit un contrat d'assurance garantissant sa responsabilité civile et celle de tout intervenant auprès de la compagnie HDE Global SE (Contrat n° 0100775930012 190006).
- Cette recherche a reçu l'avis favorable du Comité de Protection des Personnes Sud-Ouest Outre-Mer 2 le 04/07/2019. La recherche a aussi reçu l'autorisation de l'ANSM (Agence Nationale de Sécurité du Médicaments et des Produits de Santé), le 25/06/2019.

<u>AU BESOIN, QUI PUIS-CONTACTER :</u>

Pour toute question concernant l'étude, retrait de consentement, ou pour exercer vos droits concernant vos données (accès, rectification, etc...) :

L'investigateur coordonnateur de la recherche :

Dr Nicolas Grillot

✉ 1 place Alexis Ricordeau 44093 Nantes cedex 1

☎ 02 40 08 73 81

Pour toute question générale sur le traitement de vos données :

Le promoteur de la recherche, responsable du traitement :

CHU de Nantes, direction de la recherche

5 allée de l'Ile Gloriette, 44093 NANTES Cedex 1

Le Délégué à la Protection des Données (DPO) :

vosdonneespersonnelles@chu-nantes.fr

Évaluation du REMIFENTANIL en remplacement du curare pour l'induction anesthésique en séquence rapide chez le patient à risque d'inhalation de liquide gastrique- Étude multicentrique, prospective, contrôlée, randomisée, en simple aveugle, de non infériorité
Etude REMICrush
Promoteur : CHU de Nantes
N°EudraCT : 2019-000753-31

Madame, Monsieur,

Nous vous proposons de poursuivre votre participation à une recherche interventionnelle nommée REMICrush. Avant de décider si vous acceptez d'y prendre part, merci de lire soigneusement les informations qui suivent. Nous sommes également là pour répondre à toutes vos questions.

Vous avez été pris(e) en charge au bloc opératoire (ou en salle de déchocage) pour une urgence chirurgicale ou endoscopique suspectée ou avérée. Afin d'assurer votre confort, une anesthésie générale a été nécessaire. Durant l'anesthésie, afin de protéger vos poumons d'une inhalation de liquide gastrique, une sonde d'intubation a été placée dans votre trachée de manière « rapide ». Les risques de cette procédure anesthésique sont minimes mais non nuls. Ils sont en partie dus aux médicaments utilisés pour vous faire dormir « rapidement ». Classiquement nous injectons deux produits par voie intraveineuse : un hypnotique qui induit le sommeil et un curare (le SUXAMETHONIUM ou le ROCURONIUM) qui est un relaxant musculaire puissant servant à faciliter l'introduction de la sonde d'intubation dans la trachée. Cependant, les curares peuvent être responsables d'effets secondaires imprévisibles et potentiellement graves (notamment allergiques).

Le REMIFENTANIL est un morphinique déjà utilisé pour l'induction anesthésique depuis plus de 10 ans. Le REMIFENTANIL est de plus en plus fréquemment utilisé chez l'adulte et chez l'enfant à la place des curares car il n'expose pas au risque d'allergie mais son efficacité n'a pas été démontrée de façon définitive. Nous souhaitons confirmer que le REMIFENTANIL procure des conditions de mise en place de la sonde d'intubation aussi bonne que les curares.

Durant la période pré-anesthésique, nous vous avons fait participer à une étude visant à démontrer cette équivalence. Vous n'étiez pas en état d'exprimer votre volonté et vous avez été inclus(e) après procédure d'urgence. Avant d'exprimer votre accord de poursuite de participation ou votre refus de poursuite, merci de lire les informations suivantes. Vous pouvez refuser votre participation à cette étude sans aucun préjudice pour votre prise en charge.

QUEL EST L'OBJECTIF DE CETTE ETUDE

L'objectif de cette étude est de comparer l'utilisation du REMIFENTANIL à celle des curares dans l'anesthésie en urgence. Le promoteur de cette recherche est le CHU de Nantes, c'est à dire qu'il en est responsable et qu'il l'organise.

QUELS SONT LES BENEFICES ATTENDUS ET LES RISQUES POTENTIELS

Nous faisons l'hypothèse que l'utilisation du REMIFENTANIL lors de l'anesthésie générale en urgence procure des conditions d'intubation similaires aux curares, et qu'elle est mieux tolérée. En effet, bien que l'utilisation des curares pour l'intubation fasse référence depuis les années 1970, leur administration peut être responsable d'évènements indésirables imprévisibles et potentiellement graves notamment allergiques.

Le REMIFENTANIL est un morphinique utilisé depuis le début des années 2000 pour l'induction et l'entretien de l'anesthésie générale. Utilisé dans ses indications habituelles, la tolérance de ce traitement est bonne. Ses principaux effets indésirables sont ceux des morphiniques, à savoir chute de la pression artérielle, ralentissement du rythme cardiaque, ralentissement de la fréquence respiratoire, nausées, vomissements, et dans de rares cas une rigidité thoracique. Si jamais l'un de ces effets secondaires est arrivé au cours de votre anesthésie, le traitement approprié a été mis en place immédiatement.

Si vous participez à cette recherche, et que vous avez reçu le REMIFENTANIL, votre risque de développer une réaction allergique grave ou un trouble métabolique (augmentation de la quantité de potassium dans le sang) a pu être diminué en comparaison des patients recevant des curares mais il se pourrait éventuellement que la mise en place la sonde d'intubation trachéale ait été plus difficile.

METHODOLOGIE, CONTRAINTES ET DUREE DE L'ETUDE

La recherche est réalisée en collaboration avec plusieurs autres centres hospitaliers en France. Il est prévu d'inclure au total 1150 patients non à jeun pour lesquels une chirurgie sous anesthésie générale en urgence est prévue.

Comme les données médicales disponibles actuellement sont insuffisantes pour affirmer notre hypothèse, l'évaluation du REMIFENTANIL est faite par comparaison avec l'injection d'un curare.

Ainsi, les patients ayant donné leur accord pour participer à cette étude sont affectés à l'un des deux groupes de traitement par tirage au sort (randomisation) : la moitié des patients recevra la procédure classique d'induction en séquence rapide pour mise en place de la sonde d'intubation qui associe un hypnotique et le curare. L'autre moitié recevra la procédure à l'étude qui associe un hypnotique et le REMIFENTANIL. Une comparaison des deux groupes sera faite en fin d'étude.

Si la procédure intégrant le REMIFENTANIL vous a été attribuée, et que lors de l'anesthésie votre médecin a jugé nécessaire d'utiliser un curare en raison d'une situation particulière, il a très bien pu le faire.

Différentes informations ont été recueillies au cours de la procédure : pouls, tension artérielle, taux d'oxygène dans le sang, mesures des différents temps (délai de perte de conscience, durée d'intubation…) et surtout la qualité de mise en place de la sonde d'intubation.

Il n'y a pas eu et il n'y aura pas de prise de sang supplémentaire pour cette recherche.

La durée totale de votre suivi se limitera aux 7 jours suivant votre chirurgie sans que cela ne modifie votre durée de séjour à l'hôpital.

QUE SE PASSERA-T-IL A LA FIN DE LA RECHERCHE, SI LA RECHERCHE S'ARRETE ?

La recherche peut être interrompue à tout moment:

- par les autorités de santé,
- du fait du promoteur, le CHU de Nantes : si un élément nouveau survient, l'investigateur en sera informé et il vous transmettra alors les éléments susceptibles de modifier votre participation.
- du fait de l'investigateur, pour des raisons médicales vous concernant : il a pu décider à tout moment d'arrêter l'administration du produit à l'étude (par exemple à cause d'un effet secondaire ou d'une évolution de votre état de santé) et vous en informera.

Quelle que soit la raison de l'interruption, cela n'impactera d'aucune manière votre prise en charge actuelle ou future.

QUE SE PASSERA-T-IL A LA FIN DE LA RECHERCHE, SI VOUS DECIDEZ D'INTERROMPRE VOTRE PARTICIPATION ?

Si vous décidez de participer à cette recherche, il s'agira d'un acte volontaire. Vous pourrez à tout moment décider d'arrêter votre participation, sans pénalité ni préjudice. Dans ce cas, vous devez informer l'investigateur de votre décision.

Quelle que soit la raison de votre interruption, cela n'impactera d'aucune manière votre prise en charge actuelle ou future du fait du délai extrêmement court entre l'administration du traitement de l'étude et son effet,

Dans tous les cas, la qualité de votre prise en charge ne sera pas diminuée.

QUELS SONT VOS DROITS PENDANT LA RECHERCHE ?

❖ SECRET PROFESSIONNEL

Le personnel impliqué dans la recherche est soumis au secret professionnel, tout comme votre médecin traitant.

Sauf avis contraire de votre part, votre médecin traitant pourra être informé de votre participation.

❖ ACCES AUX DONNEES VOUS CONCERNANT - TRAITEMENT DES DONNEES - CONFIDENTIALITE

Dans le cadre de cette recherche, un traitement informatique de vos données personnelles va être mis en œuvre : cela permettra d'analyser les résultats de la recherche et de remplir l'objectif de la recherche.

Pour cela, les données médicales vous concernant ont été et seront recueillies dans un cahier électronique (eCRF).

Afin d'assurer leur confidentialité, ces données seront identifiées par un numéro de code et vos initiales.

L'ensemble de ces données sera transmis au Promoteur de la recherche (CHU de Nantes).

Ces données pourront également, dans des conditions assurant leur confidentialité, être transmises aux autorités sanitaires habilitées.

Elles seront susceptibles d'être exploitées dans le cadre de publications ou de communications; dans ce cas, votre anonymat sera préservé.

Si vous décidez de ne pas donner votre consentement pour participer à la recherche, les données obtenues avant votre décision de ne pas poursuivre la recherche seront utilisées. Les données recueillies après le retrait de votre consentement ne seront pas utilisées pour cette recherche et resteront destinées à l'usage strict du soin.

Conformément aux dispositions de la loi relative à l'informatique aux fichiers et aux libertés (loi modifiée du 6 janvier 1978), de la loi n° 2018-493 du 20 juin 2018 relative à la protection des données personnelles et du Règlement (UE) 2016/679 du Parlement européen et du Conseil du 27 avril 2016 relatif à la protection des personnes physiques à l'égard du traitement des données à caractère personnel et à la libre circulation de ces données (RGPD), vous disposez d'un droit d'accès, de rectification, et de limitation du traitement de vos données personnelles. Vous pouvez également porter une réclamation auprès d'une autorité de contrôle (CNIL pour la France : https://www.cnil.fr/fr/webform/adresser-une-plainte/).

Ces données pourront être utilisées lors de recherches ultérieures exclusivement à des fins scientifiques. Vous pouvez retirer votre consentement à cette utilisation ultérieure ou exercer votre faculté d'opposition à tout moment.

Vous pouvez également accéder directement ou par l'intermédiaire d'un médecin de votre choix à l'ensemble de vos données médicales en application des dispositions de l'article L 1111-7 du Code de la Santé Publique.

Vos données seront conservées tout au long de la recherche. Après la fin de la recherche, les données seront archivées pour une durée conforme aux dispositions réglementaires, puis détruites.

Pour en savoir plus ou exercer vos droits concernant vos données, voir vos contacts ci-dessous.

❖ ACCES AUX RESULTATS GLOBAUX DE LA RECHERCHE

A la fin de la recherche, et à votre demande, vous pourrez être informé(e) par l'investigateur des résultats globaux de cette recherche (dès qu'ils seront disponibles).

QUELLES SONT VOS OBLIGATIONS PENDANT LA RECHERCHE ?

❖ VOS OBLIGATIONS

Vous devez informer l'investigateur de tous les médicaments que vous prenez.

Vous devez aussi l'informer immédiatement de tout évènement ou effet indésirable éventuellement rencontré au cours de votre participation à la recherche.

❖ PROTECTION SOCIALE

Pour pouvoir participer à cette recherche vous devez être affilié(e) ou bénéficier d'un régime de sécurité sociale

❖ MODALITES DE PARTICIPATION A UNE AUTRE RECHERCHE

Vous ne pourrez pas participer à une autre recherche interventionnelle pendant toute la durée de votre participation à la Recherche c'est-à-dire 24 heures après votre chirurgie.

QUEL EST LE CADRE REGLEMENTAIRE DE CETTE RECHERCHE ?

Cette recherche est conforme :

- Aux articles L. 1121-1 à L. 1126-12 du code de la santé publique relatifs aux recherches impliquant la personne humaine

- A la loi « Informatique et Libertés » du 6 janvier 1978 modifiée et la loi n° 2018-493 du 20 juin 2018 relative à la protection des données personnelles

- au Règlement (UE) 2016/679 du Parlement européen et du Conseil du 27 avril 2016 relatif à la protection des personnes physiques à l'égard du traitement des données à caractère personnel et à la libre circulation de ces données (RGPD)

Vous pouvez retrouver tous ces textes sur le site http://www.legifrance.gouv.fr

Conformément aux dispositions réglementaires :

➢ Le CHU de Nantes organise cette recherche en tant que « promoteur ». Il a souscrit un contrat d'assurance garantissant sa responsabilité civile et celle de tout intervenant auprès de la compagnie HDE Global SE (Contrat n° 0100775930012 190006).

➢ Cette recherche a reçu l'avis favorable du Comité de Protection des Personnes Sud-Ouest Outre-Mer 2 le 04/07/2019. La recherche a aussi reçu l'autorisation de l'ANSM (Agence Nationale de Sécurité du Médicaments et des Produits de Santé), le 25/06/2019.

AU BESOIN, QUI PUIS-CONTACTER :

Pour toute question concernant l'étude, retrait de consentement, ou pour exercer vos droits concernant vos données (accès, rectification, etc...) :

L'investigateur coordonnateur de la recherche :

Dr Nicolas Grillot

✉ 1 place Alexis Ricordeau 44093 Nantes cedex 1

☎ 02 40 08 73 81

Pour toute question générale sur le traitement de vos données :

Le promoteur de la recherche, responsable du traitement :

CHU de Nantes, direction de la recherche

5 allée de l'Ile Gloriette, 44093 NANTES Cedex 1

Le Délégué à la Protection des Données (DPO) :

vosdonneespersonnelles@chu-nantes.fr

CENTRE HOSPITALIER UNIVERSITAIRE DE NANTES	**Attestation de consentement à une recherche REMICrush**<br>« Évaluation du REMIFENTANIL en remplacement du curare pour l'induction anesthésique en séquence rapide chez le patient à risque d'inhalation de liquide gastrique »<br>**Promoteur *: CHU de Nantes***
**Version n° 1.0**<br>**Date : 25/04/2019**	***Ref : RC19_0055 - N° EudraCT 2019-000753-31***

Je soussigné(e)

M^{e}, M. (*rayer la mention inutile*) (*prénom*, NOM) ………………………………………………………………………….
……,

Date de naissance : …../……/……..
accepte librement et volontairement de participer à la recherche référencée ci-dessus, coordonnée par le Docteur Nicolas GRILLOT et organisée par le CHU de Nantes, promoteur de la recherche.

Etant entendu que :

- L'investigateur qui m'a informé(e) et a répondu clairement à toutes mes questions, m'a précisé que ma participation est libre et que je peux me retirer de la recherche à tout moment.
- J'atteste ne pas faire l'objet de mesure de protection (tutelle, curatelle, sauvegarde de justice), en outre je confirme être affilié(e) ou bénéficier d'un régime de sécurité sociale.
- Il m'a été préalablement remis une note d'information sur cette recherche précisant son but, sa méthodologie, ses bénéfices attendus et ses risques prévisibles.
- Je pourrai avoir communication par l'investigateur, au cours ou à l'issue de la recherche, des informations qu'il détient concernant ma santé.
- Je suis parfaitement conscient(e) que je peux retirer à tout moment mon consentement à ma participation à cette recherche et cela quelles que soient mes raisons et sans supporter aucune responsabilité, mais je m'engage dans ce cas à en informer l'investigateur. Le fait de ne plus participer à cette recherche ne portera pas atteinte à mes relations avec cet investigateur, ni à la qualité des soins qui me seront donnés.
- J'accepte que mon médecin traitant soit informé de ma participation à la recherche :

  ☐ Oui, j'accepte ☐ Non, je refuse
- Je pourrai à tout moment demander des informations complémentaires à l'investigateur.
- Si je le souhaite, à son terme, je serai informé(e) par l'investigateur des résultats globaux de cette recherche.
- Mon consentement ne décharge en rien l'investigateur et le promoteur de l'ensemble de leurs responsabilités et je conserve tous mes droits garantis par la loi.
- Je ne pourrai pas participer à une autre recherche interventionnelle pendant les 24 heures suivant mon intervention chirurgicale.
- J'accepte que les données enregistrées à l'occasion de cette recherche puissent faire l'objet d'un traitement informatisé par le promoteur ou pour son compte, et j'atteste avoir été informé de tous mes droits concernant mes données personnelles selon les modalités décrites dans la note d'information en vigueur, qui m'a été transmise pour ce protocole.

**PERSONNE SE PRETANT A LA RECHERCHE**		
**Date :**<br>**...... / ..... / ………..**	**Signature :**	
Attestation du consentement en cas d'impossibilité d'expression écrite de la personne qui se prête à la recherche par la personne de confiance, un membre de la famille ou un des proches		
Date :<br>...... / ..... / ………..	Prénom NOM :<br>Lien avec la personne de prêtant à la recherche :	Signature :

**INVESTIGATEUR : J'atteste avoir pleinement expliqué à la personne signataire le but, les modalités ainsi que les risques potentiels de la recherche**		
**Date :**<br>**...... / ..... / ………..**	**NOM :**	**Signature :**

Ce document est à réaliser en 2 exemplaires originaux : le premier doit être conservé par l'investigateur et le deuxième est remis à la personne donnant son consentement. En cas de duplicata, l'original est conservé par l'investigateur et une copie est remise à la personne ayant donné son consentement. En cas de triplicata, le promoteur récupèrera un des duplicata des consentements dans des enveloppes scellées tout au long de l'étude.

CENTRE HOSPITALIER UNIVERSITAIRE DE NANTES	**Attestation de consentement à une recherche en situation d'urgence pour un majeur hors d'état d'exprimer son consentement**<br>**(Personne de confiance ou proche)**<br>**REMICrush**<br>« Évaluation du REMIFENTANIL en remplacement du curare pour l'induction anesthésique en séquence rapide chez le patient à risque d'inhalation de liquide gastrique »<br>**Promoteur *: CHU de Nantes***
**Version n° 1.0**<br>**Date : 25/04/2019**	***Ref : RC19_0055 - N° EudraCT 2019-000753-31***

Je soussigné(e), M[e], M. *(rayer la mention inutile)* (*prénom*, *nom*)…………………………………………………

représentant du patient M[e], M. *(rayer la mentions inutile)* (*prénom*, *nom*)………………………………………….

signataire en tant que : ☐ personne de confiance ☐ membre de la famille ou proche

J'accepte librement et volontairement que le patient représenté participe à la recherche référencée ci-dessus, coordonnée par le Docteur Nicolas GRILLOT et organisée par le CHU de Nantes, promoteur.

Etant entendu que :

- Son état le permettant, le patient représenté a été consulté et n'a pas exprimé de refus
- L'investigateur qui m'a informé(e) et a répondu clairement à toutes mes questions, m'a précisé que la participation du patient représenté est libre et que je peux retirer mon accord sur sa participation à tout moment.
- Il m'a été remis une note d'information sur cette recherche précisant son but, sa méthodologie, ses bénéfices attendus et ses risques prévisibles.
- J'atteste que le patient représenté ne fait pas l'objet de mesure de protection sous sauvegarde de justice, en outre je confirme qu'il/elle est affilié(e) ou bénéficie d'un régime de sécurité sociale.
- En situation d'urgence : le patient représenté sera informé et son consentement recherché dès que cela sera possible
- Je pourrai avoir communication par l'investigateur, au cours ou à l'issue de la recherche, des informations qu'il détient concernant la santé du patient représenté.
- Je suis parfaitement conscient(e) que je peux arrêter la participation du patient représenté à cette recherche et cela quelles que soient mes raisons et sans supporter aucune responsabilité, mais je m'engage dans ce cas à en informer l'investigateur. Le fait de ne plus participer à cette recherche ne portera pas atteinte aux relations avec l'investigateur, ni à la qualité des soins qui seront donnés au patient représenté.
- J'accepte que le médecin traitant du patient soit informé de sa participation à la recherche :
  ☐ Oui, j'accepte ☐ Non, je refuse
- Je pourrai à tout moment demander des informations complémentaires à l'investigateur.
- Si je le souhaite, à son terme, je serai informé(e) par l'investigateur des résultats globaux de cette recherche.
- Mon consentement ne décharge en rien l'investigateur et le promoteur de l'ensemble de leurs responsabilités et le patient représenté conserve tous ses droits garantis par la loi.
- Le patient représenté ne pourra pas participer à une autre recherche interventionnelle pendant les 24 heures suivant son intervention chirurgicale.
- J'accepte que les données enregistrées à l'occasion de cette recherche puissent faire l'objet d'un traitement informatisé par le promoteur ou pour son compte, et j'atteste avoir été informé de tous les droits concernant ces données personnelles selon les modalités décrites dans la note d'information en vigueur, qui m'a été transmise pour ce protocole.

**REPRESENTANT DU PATIENT**	
**Date :**<br>**...... / ..... / ………..**	**Signature :**

**INVESTIGATEUR : J'atteste avoir pleinement expliqué à la personne signataire le but, les modalités ainsi que les risques potentiels de la recherche**		
**Date :**<br>**...... / ..... / ………..**	**NOM :**	**Signature :**

Ce document est à réaliser en 2 exemplaires originaux : le premier doit être conservé par l'investigateur et le deuxième est remis à la personne donnant son consentement. En cas de duplicata, l'original est conservé par l'investigateur et une copie est remise à la personne ayant donné son consentement. En cas de triplicata, le promoteur récupèrera un des duplicata des consentements dans des enveloppes scellées tout au long de l'étude.

Évaluation du REMIFENTANIL en remplacement du curare pour l'induction anesthésique en séquence rapide chez le patient à risque d'inhalation de liquide gastrique- Étude multicentrique, prospective, contrôlée, randomisée, en simple aveugle, de non infériorité
Etude REMICrush
Promoteur : CHU de Nantes
N°EudraCT : 2019-000753-31

Madame, Monsieur,

En raison de son état qui le/la rend incapable d'exprimer son consentement, devant l'urgence de la situation et conformément à la loi, c'est à vous que nous demandons l'autorisation de participation de la personne que vous représentez à cette recherche interventionnelle nommée REMICrush.
Avant de vous décider, merci de lire soigneusement les informations qui suivent. Nous sommes également là pour répondre à toutes vos questions d'ici avant que nous ne débutions sa prise en charge anesthésique.

Son état de santé actuel nécessite une intervention chirurgicale ou endoscopique urgente sous anesthésie générale. Les risques de cette anesthésie sont minimes mais non nuls. Ils sont en partie dus aux médicaments utilisés pour le/la faire dormir mais également au fait qu'il/elle ait mangé dans les 12 heures précédant son intervention chirurgicale (non-respect du jeun pré-opératoire).

Pour réaliser une anesthésie générale dans ce contexte, il est recommandé d'administrer deux produits par voie intraveineuse :

- un hypnotique qui induit le sommeil
- un curare (le SUXAMETHONIUM ou le ROCURONIUM) qui est un relaxant musculaire puissant servant à faciliter l'introduction rapide de la sonde d'intubation dans la trachée (ce qui servira à assurer son oxygénation et protégera ses poumons d'une éventuelle inhalation de liquide gastrique durant l'intervention).

Les curares peuvent être responsables d'effets secondaires imprévisibles et potentiellement graves (notamment allergiques). Le REMIFENTANIL est un morphinique déjà utilisé pour l'induction anesthésique depuis plus de 10 ans et qui est de plus en plus fréquemment utilisé à la place des curares car il n'expose pas au risque d'allergie.

Nous souhaitons confirmer que le REMIFENTANIL permet la mise en place de la sonde d'intubation dans d'aussi bonnes conditions que les curares dans le contexte du non-respect du jeûn pré-opératoire.

QUEL EST L'OBJECTIF DE CETTE ETUDE

L'objectif de cette étude est de comparer l'utilisation du REMIFENTANIL à celle des curares dans l'anesthésie en urgence. Le promoteur de cette recherche est le CHU de Nantes, c'est-à-dire qu'il en est responsable et qu'il l'organise.

QUELS SONT LES BENEFICES ATTENDUS ET LES RISQUES POTENTIELS

Nous faisons l'hypothèse que l'utilisation du REMIFENTANIL lors de l'anesthésie générale en urgence procure des conditions d'intubation similaires aux curares, et qu'elle est mieux tolérée. En effet, bien que l'utilisation des curares pour l'intubation fasse référence depuis les années 1970, leur administration peut être responsable d'évènements indésirables imprévisibles et potentiellement graves notamment allergiques.

Le REMIFENTANIL est un morphinique utilisé depuis le début des années 2000 pour l'induction et l'entretien de l'anesthésie générale. Utilisé dans ses indications habituelles, la tolérance de ce traitement est bonne. Ses principaux effets indésirables sont ceux des morphiniques, à savoir chute de la pression artérielle,

ralentissement du rythme cardiaque, ralentissement de la fréquence respiratoire, nausées, vomissements, et dans de rares cas une rigidité thoracique. Si toutefois l'un de ces effets secondaires survenait, le traitement approprié serait mis en place immédiatement.

Si la personne que vous représentez participait à cette recherche, et qu'elle recevait le REMIFENTANIL, son risque de développer une réaction allergique grave ou un trouble métabolique (augmentation de la quantité de potassium dans le sang) pourrait être diminué en comparaison des patients qui recevront des curares mais il pourrait également être plus difficile de mettre en place la sonde d'intubation trachéale.

METHODOLOGIE, CONTRAINTES ET DUREE DE L'ETUDE

La recherche est réalisée en collaboration avec plusieurs autres centres hospitaliers en France. Il est prévu d'inclure au total 1150 patients non à jeun pour lesquels une chirurgie sous anesthésie générale en urgence est prévue.

Comme les données médicales disponibles actuellement sont insuffisantes pour affirmer notre hypothèse, l'évaluation du REMIFENTANIL se fera par comparaison avec l'injection d'un curare.

Ainsi, chaque patient qui participera à cette étude sera affecté à l'un des deux groupes de traitement par tirage au sort (randomisation): la moitié des patients recevra la procédure classique associant un hypnotique et le curare et l'autre moitié recevra la procédure à l'étude associant un hypnotique et le REMIFENTANIL. Une comparaison des deux groupes sera faite en fin d'étude.

Si la procédure intégrant le REMIFENTANIL était attribuée à la personne que vous représentez, et que lors de l'anesthésie votre médecin jugeait nécessaire d'utiliser un curare en raison d'une situation particulière, il pourra tout à fait le faire.

Différentes informations seront recueillies au cours de la procédure : pouls, tension artérielles, taux d'oxygène dans le sang, mesures des différents temps (délai de perte de conscience, durée d'intubation…) et surtout la qualité de mise en place de la sonde d'intubation.

Il n'y a pas de prise de sang supplémentaire pour cette recherche.

La durée totale de son suivi se limitera aux 7 jours suivant la chirurgie sans que cela ne modifie la durée de son séjour à l'hôpital.

QUE SE PASSERA-T-IL A LA FIN DE LA RECHERCHE, SI LA RECHERCHE S'ARRETE ?

La recherche peut être interrompue à tout moment:

- par les autorités de santé,
- du fait du promoteur, le CHU de Nantes : si un élément nouveau survient, l'investigateur en sera informé et il vous transmettra alors les éléments susceptibles de modifier votre participation.
- du fait de l'investigateur, pour des raisons médicales vous concernant : il peut décider à tout moment d'arrêter l'administration du produit à l'étude (par exemple à cause d'un effet secondaire ou d'une évolution de l'état de santé de la personne que vous représentez) et vous en informera ou informera directement la personne représentée.

Quelle que soit la raison de l'interruption, l'investigateur vous informera ou informera directement la personne représentée alors des mesures à suivre. Du fait du délai extrêmement court entre l'administration de la procédure à l'étude et son effet, l'interruption de la recherche n'impactera d'aucune manière sa prise en charge actuelle ou future.

QUE SE PASSERA-T-IL A LA FIN DE LA RECHERCHE, SI VOUS DECIDEZ D'INTERROMPRE VOTRE PARTICIPATION ?

Si vous acceptez que la personne que vous représentez participe à cette recherche, il s'agira d'un acte volontaire. Vous pourrez à tout moment, tant qu'elle sera incapable s'exprimer son consentement ou son

opposition, décider d'arrêter sa participation, sans pénalité ni préjudice. Dans ce cas, vous devez informer l'investigateur de votre décision.

Quelle que soit la raison de l'interruption, aucune mesure supplémentaire ne sera prise car la personne que vous représentez aura déjà reçu la procédure à l'étude.

Dans tous les cas, la qualité de sa prise en charge ne sera pas diminuée.

QUELS SONT VOS DROITS PENDANT LA RECHERCHE ?

- SECRET PROFESSIONNEL

Le personnel impliqué dans la recherche est soumis au secret professionnel, tout comme le médecin traitant de la personne que vous représentez.

- ACCES AUX DONNEES VOUS CONCERNANT - TRAITEMENT DES DONNEES - CONFIDENTIALITE

Dans le cadre de cette recherche, un traitement informatique des données personnelles de la personne que vous représentez va être mis en œuvre : cela permettra d'analyser les résultats de la recherche et de remplir l'objectif de la recherche.

Pour cela, les données médicales le/la concernant seront recueillies dans un cahier électronique (eCRF).

Afin d'assurer leur confidentialité, ces données seront identifiées par un numéro de code et ses initiales.

L'ensemble de ces données sera transmis au Promoteur de la recherche (CHU de Nantes).

Ces données pourront également, dans des conditions assurant leur confidentialité, être transmises aux autorités sanitaires habilitées.

Elles seront susceptibles d'être exploitées dans le cadre de publications ou de communications; dans ce cas, son anonymat sera préservé.

Si vous décidez de retirer votre consentement pour la participation à la recherche de la personne que vous représentez, les données obtenues avant que celui-ci n'ait été retiré seront utilisées. Les données recueillies après le retrait de votre consentement ne seront pas utilisées pour cette recherche et resteront destinées à l'usage strict du soin.

Conformément aux dispositions de la loi relative à l'informatique aux fichiers et aux libertés (loi modifiée du 6 janvier 1978), de la loi n° 2018-493 du 20 juin 2018 relative à la protection des données personnelles et du Règlement (UE) 2016/679 du Parlement européen et du Conseil du 27 avril 2016 relatif à la protection des personnes physiques à l'égard du traitement des données à caractère personnel et à la libre circulation de ces données (RGPD), vous disposez d'un droit d'accès, de rectification, et de limitation du traitement de vos données personnelles. Vous pouvez également porter une réclamation auprès d'une autorité de contrôle (CNIL pour la France : https://www.cnil.fr/fr/webform/adresser-une-plainte/).

Ces données pourront être utilisées lors de recherches ultérieures exclusivement à des fins scientifiques. Vous pouvez retirer votre consentement à cette utilisation ultérieure ou exercer votre faculté d'opposition à tout moment.

Les données seront conservées tout au long de la recherche. Après la fin de la recherche, les données seront archivées pour une durée conforme aux dispositions réglementaires, puis détruites.

Pour en savoir plus ou exercer vos droits concernant vos données, voir vos contacts ci-dessous.

❖ ACCES AUX RESULTATS GLOBAUX DE LA RECHERCHE

A la fin de la recherche, et à votre demande, vous et la personne que vous représentez pourrez être informé(e)s par l'investigateur des résultats globaux de cette recherche (dès qu'ils seront disponibles).

QUELLES SONT VOS OBLIGATIONS PENDANT LA RECHERCHE ?

❖ VOS OBLIGATIONS

Si vous en avez connaissance :

- Vous devez informer l'investigateur de tous les médicaments que la personne que vous représentez prend.

- Vous devez aussi l'informer immédiatement de tout évènement ou effet indésirable éventuellement rencontré par la personne que vous représentez au cours de sa participation à la recherche.

❖ PROTECTION SOCIALE

Pour pouvoir participer à cette recherche la personne que vous représentez doit être affilié(e) ou bénéficier d'un régime de sécurité sociale.

❖ MODALITES DE PARTICIPATION A UNE AUTRE RECHERCHE

La personne que vous représentez ne pourra pas participer à une autre recherche interventionnelle pendant toute la durée de sa participation à la Recherche c'est-à-dire 24 heures après sa chirurgie.

QUEL EST LE CADRE REGLEMENTAIRE DE CETTE RECHERCHE ?

Cette recherche est conforme :

- Aux articles L. 1121-1 à L. 1126-12 du code de la santé publique relatifs aux recherches impliquant la personne humaine

- A la loi « Informatique et Libertés » du 6 janvier 1978 modifiée et la loi n° 2018-493 du 20 juin 2018 relative à la protection des données personnelles

- au Règlement (UE) 2016/679 du Parlement européen et du Conseil du 27 avril 2016 relatif à la protection des personnes physiques à l'égard du traitement des données à caractère personnel et à la libre circulation de ces données (RGPD)

Vous pouvez retrouver tous ces textes sur le site http://www.legifrance.gouv.fr

Conformément aux dispositions réglementaires :

➢ Le CHU de Nantes organise cette recherche en tant que « promoteur ». Il a souscrit un contrat d'assurance garantissant sa responsabilité civile et celle de tout intervenant auprès de la compagnie HDE Global SE (Contrat n° 0100775930012 190006).

➢ Cette recherche a reçu l'avis favorable du Comité de Protection des Personnes Sud-Ouest Outre-Mer 2 le 04/07/2019. La recherche a aussi reçu l'autorisation de l'ANSM (Agence Nationale de Sécurité du Médicaments et des Produits de Santé), le 25/06/2019.

AU BESOIN, QUI PUIS-CONTACTER :

Pour toute question concernant l'étude, retrait de consentement, ou pour exercer vos droits concernant vos données (accès, rectification, etc...) :

L'investigateur coordonnateur de la recherche :

Dr Nicolas Grillot

🖂 1 place Alexis Ricordeau 44093 Nantes cedex 1

☎ 02 40 08 73 81

Pour toute question générale sur le traitement de vos données :

Le promoteur de la recherche, responsable du traitement :

CHU de Nantes, direction de la recherche

5 allée de l'Ile Gloriette, 44093 NANTES Cedex 1

Le Délégué à la Protection des Données (DPO) :

vosdonneespersonnelles@chu-nantes.fr

Je soussigné(e) Docteur ………………………………………………………………………….

Investigateur dans l'étude REMICRUSH certifie avoir examiné le patient

Monsieur / Madame …………………………………………………………………………….

afin d'évaluer son aptitude à signer le formulaire de consentement de l'étude.

Du fait de sa maladie, le patient est incapable à l'heure actuelle de comprendre les informations concernant l'étude REMICRUSH à laquelle nous lui proposons de participer et de donner personnellement son consentement.

Dans cette situation d'urgence au vu des contraintes dues au délai d'inclusion, il n'est également pas possible d'obtenir un consentement de la personne de confiance ou d'un membre de la famille.

Je m'engage conformément à la loi (L1122-1-2 du Code de Santé Publique) à informer le patient, et si possible la personne de confiance ou un membre de la famille, et à rechercher un consentement rétrospectif dès que possible.

**INVESTIGATEUR :**		
**Date :**<br>**...... / ..... / ………..**	**NOM :**	**Signature :**
